# Editorial: Biobased nanomaterials: New trends and applications 

**DOI:** 10.3389/fchem.2022.1028321

**Published:** 2022-10-13

**Authors:** Francisco J. Martin-Martinez, Jingjie Yeo, James W. Ryan, Antoni Forner-Cuenca, Maria-Magdalena Titirici

**Affiliations:** ^1^ Department of Chemistry, Swansea, United Kingdom; ^2^ Sibley School of Mechanical and Aerospace Engineering, College of Engineering, Cornell University, Ithaca, NY, United States; ^3^ Department of Chemical Engineering and Chemistry, Eindhoven University of Technology, Eindhoven, Netherland; ^4^ Department of Chemical Engineering, Faculty of Engineering, Imperial College London, London, United Kingdom

**Keywords:** biobased, nanomaterials, biomass, bioeconomy, carbon materials

**FIGURE 1:**
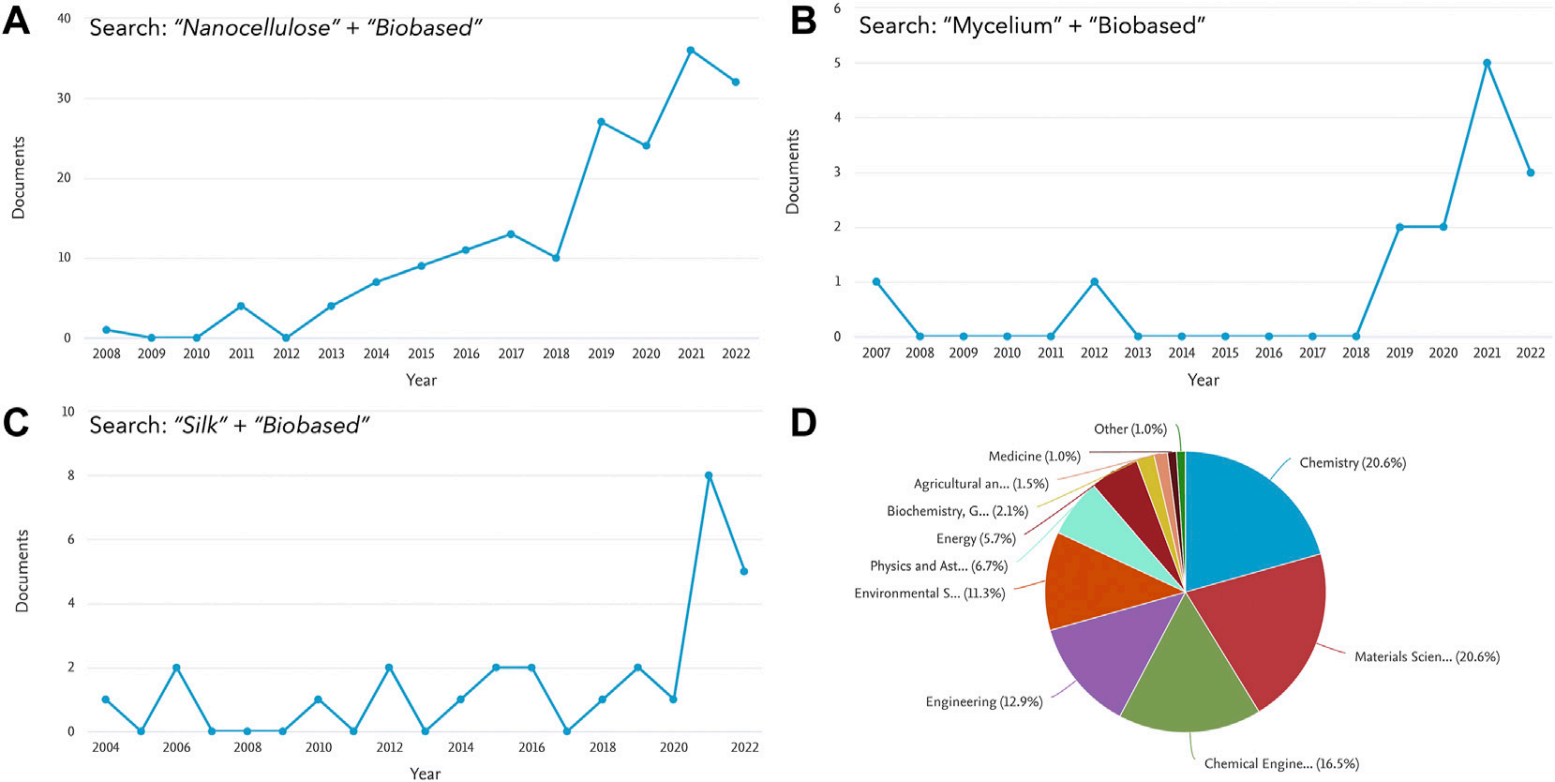
Brief statical analysis showing the contribution of some of the reported biobased materials, **(A)** number of publications from 2008 to 2022 including the keyworks nanocellulose and biobased on their title, abstract and keywords section, **(B)** number of publications from 2008 to 2022 including the keyworks mycelium and biobased on their title, abstract and keywords section, **(C)** number of publications from 2008 to 2022 including the keyworks silk and biobased on their title, abstract and keywords section, **(D)** analysis of publications by scientific area from 2008 to 2022 including the keyworks biobased nanomaterials on their title, abstract and keywords section.

The conjunction of world’s limited natural resources and the ever-growing material needs of our increasing global population is environmentally and economically unsustainable. Subsequently, the impact of climate change, the depletion of fossil fuels, increasing social pressures, and the implementation of new policies across the globe, are slowly driving our society towards a biobased economy that unlocks the full potential of all types of sustainably sourced biomass, crop residues, industrial side-streams and wastes to produce value-added products (Lange et al., 2021). The complexity of this transition to a new economic model is substantial, and it challenges scientists, policy makers, and industry platforms. Nevertheless, our society is starting to rethink material and energy sources, supply chains, and product design across industries. Therefore, as this biobased economy develops and a new alternative model emerges, it is paramount to advance in sustainable material, and energy production, identifying new applications, and determining possible impacts of this technological development (Weiss et al., 2012). In fact, a plethora of applications that valorize biomass (Hu et al., 2010), and implement biomass-derived (i.e., biobased) materials, are being intensively investigated. These biobased materials are specially focused on energy storage (Titirici et al., 2012), platform chemicals, biomedicine (Abdelhamid and Mathew, 2022), and char production for soil remediation (Correa and Kruse, 2018), but also include biofuels, nanomaterials, carbon fibers, adhesives, foams, and coatings, just to mention a few. Within this context, this research topic provides some new trends and applications of emerging biobased nanomaterials, including biobased carbon fibers, wood-based materials, and mycelium. As shown in Figure 1, the number of publications that include “biobased” with materials like mycelium (Grimm and Wösten, 2018), nanocellulose (Klemm et al., 2011) or silk (Vepari and Kaplan, 2007), is significantly increasing in the last years.

The topic also spotlights pertinent challenges associated with scale-up, sustainability, or material property control at the nanoscale. It provides examples that will hopefully point out relevant research directions and will also steer scientific discussion on the use of biobased nanomaterials, as standalone components or in combination with biomaterials, for applications ranging from energy storage to biomedicine, or sensing. Given the relevance that valorizing biomass sources has for the above-mentioned transition to a more sustainable development, we believe that any contribution towards understanding the potential of biobased materials in any application is mandatory. While applications of wood-based and wood-derived materials are better defined than those associated for mycelium, their scaled-up production is still challenging. In other cases, like those materials for biomedical applications, many fundamental mechanisms need to be yet understood.

In this research topic, Montanari et al. provide a perspective on new sustainable wood nanotechnologies and wood-based composite materials. Wood is by far the most abundant biomass source for biobased materials development, and the possibilities to develop wood substrates as nanomaterial components for large, load-bearing structures are countless. However, achieving large, multifunctional structures from sustainable wood nanomaterials is challenging. Although existing wood composites are already available commercially, there is still a lack of control of the material properties at the nanoscale, and we still need to identify the best directions opportunities for applications. Furthermore, to successfully achieve efficient wood-based biorefineries, it is crucial to valorize lignin, one of the components of lignocellulosic biomass, and the most abundant natural polymer after cellulose. In this direction, Thielke et al. present a sustainable electrospinning process to upscale lignin with high molecular weight into freestanding nonwoven carbon fiber electrodes for supercapacitors. This is especially relevant, given the needs for more efficient materials in energy storage.

In addition to wood, and lignocellulosic biomass, a natural material that is drawing increasing scientific attention nowadays is mycelium, the vegetative part of a fungus. As pointed out by Yan et al. mycelium has the unique capability to utilize agricultural crop waste (e.g., sugarcane bagasse, rice husks, cotton stalks, straw, and stover) as substrate for the growth of its network. During the growing process, mycelium is capable of integrating the wastes from both pieces and continuous composites without energy input, and without any generating extra waste. Furthermore, mycelium is rich in chitin, which makes it also useful for chitin-based materials. As reviewed by these authors, mycelium-based and mycelium-derived materials are being widely applied in different areas, including construction, manufacturing, agriculture, and biomedicine.

One of the most pressing challenges across industries is the development of efficient and scalable processes. Qi et al. presented a study that is focused also on carbon fibers, although in their work they have developed a process to transform silk into continuous carbon fibers by using a highly scalable one-step heating procedure without any additives or activation process. This is a lean strategy to produce carbon fibers that are electroconductive and mechanically robust, while proposing a scale-up strategy to prepare animal silk-derived amorphous carbon fibers that can potentially be extrapolated to other raw materials. Given the challenges associated to the development of efficient and scalable processes across industries, this work represents a specially interesting case study. Also on silk, Shen et al. summarizes various methods to synthesize functional silk-based materials from different perspectives. This authors also highlight the recent advances in the applications of natural and recombinant silks in tissue regeneration, soft robotics, and biosensors, using *Bombyx mori* SF and silk-elastin-like proteins as examples.

Pertaining to biobased nanomaterials for biomedical purposes, Xiang et al. summarizes the research based on the characteristics of imageable nanomaterials, while highlighting the advantages and biomedical applications of imageable nanomaterials in the diagnosis and treatment of diseases and discussing current challenges and prospects. This study is of special interest, since it provides an example of biomaterials, i.e., a material engineered to interface with biological systems, which is also biobased. Also, in the context of biomedical applications, Thomas et al. provides an update on the contemporary strategies utilizing exosomes for theranostic applications in nanomedicine. In addition, the authors provide a synopsis of exosomal features and insights into strategic modifications that control *in vivo* biodistribution. The authors further discuss the opportunities, merits, and pitfalls for cell/tissue targeted drug delivery in personalized nanotherapy. We believe that research on exosomes will dramatically increase in the years to come, given their importance of in inter- and intra-cell communication, and the many lessons that come be learnt from their study. Thus, the connection of exosomes research and biobased materials is an area that should be explored, and accordingly any initial studies along those lines are very relevant.
